# Its Site Can Accommodate 400 Beds

**Published:** 1913-06-21

**Authors:** 


### THE PRINCE OF WALES'S GENERAL HOSPITAL, TOTTENHAM.

### Its Site can Accommodate 400 Beds.

The festival dinner of this institution was held on the 11th instant at the Savoy Hotel, the Right Hon. Viscount Hill in the chair. Sir Henry Burdett, in proposing prosperity to the hospital, gave a graphic account of its evolution and his association with it during the last thirty-two years. Up to 1897 it was a Deaconess Institution with a few beds; in 1898 the Charity Commissioners formulated a scheme, and in 1899 it was changed to a voluntary hospital. Commencing in 1899 with twenty-four beds, an income of £1,200, and an expenditure of £5,000, it has become, by the energetic action of the late Sir Francis Cory-Wright, the Ladies' Committee, and others, a general hospital of 125 beds, with an ordinary income in 1912 of £8,000 and a total income of nearly £10,000. During this period £20,000 has been raised and spent upon buildings, and the hospital to-day has practically each department which collectively make up a modern hospital. What the hospital needs to enable it to fulfil its responsibilities to the huge district, containing a population of 450,000, which it serves is more income, a reasonable amount of capital—it possesses practically none at present—and more beds. He found evidence of overcrowding in some wards, which should be prevented by those responsible. This hospital has often been characterised as too economical, but where funds are scanty economy must be enforced, and the economical management of Miss Fox, the present Matron, has represented efficiency all through the institution. No one could have produced the results to be found at the Prince of Wales's Hospital, the real, solid comfort and good food, the attendance on the patients, the nursing, and all the adjuncts of a modern hospital, if this hospital had not been fortunate enough to have at the head of the domestic staff and nursing departments a lady who has a special gift for and a special knowledge of such matters. Sir Henry, who had specially inspected the hospital before making his speech, complimented the medical and general administration of the hospital, and congratulated the Secretary-Director and Committee on the interesting form in which the report of the hospital is prepared and the clearness and interest with which the statistics are set forth. Speaking of the Matron, Miss Fox, he said that in his judgment her merits entitle her to a much higher responsibility and remuneration than Tottenham can at present give her. Sir Henry spoke at length upon the material increase in the pressure upon hospital beds which must result in London during the next few years, when the National Insurance Act is put into full force. He showed that there will be a shortage in the Metropolis alone in all probability of quite 10,000 hospital beds if insured persons are to obtain at once the in-patient treatment they should receive. At Tottenham there is an urgent need for a hospital of 400 beds at least, and the Prince of Wales's General Hospital is fortunate in having an excellent site of 4½ acres on which the necessary buildings could be placed and a great hospital organised and planned, providing the necessary money is forthcoming. No more economical method of providing the hospital treatment required by the half-million of people surrounding the Prince of Wales's General Hospital could be found than by an arrangement between the County Council and the Insurance Commissioners, through the Local Government Board, upon a plan which would preserve the voluntary system, provide the necessary funds to defray the cost of the extra buildings, and also on the *pro rata* system ensure the provision of the *pro rata* cost of the treatment of each insured person dealt with in the new pavilions. The London County Council had taken up a large building site in Tottenham which had brought 20,000 new people at least into the district, and promised to bring more; the inhabitants of these County Council buildings were dependent for hospital accommodation on the Prince of Wales's Hospital, and the County Council had never contributed a penny or a single bed for their accommodation. No municipal or local authority was justified in clearing out the population in congested districts of a great city and placing them on a new site without securing them adequate provision for their hospital treatment and that of their wives and dependants. It was a great blot upon the reputation of England that politics should at the present time make social progress and wise and adequate health provision impossible, owing to the continual wrangling of party advocates on one side or the other. The inhabitants of Greater London suffered grave and avoidable miseries and incurred violent deaths at the present time owing to the failure of the London County Council to do its duty, as the Corporation of the City of London had done, by providing and equipping an adequate ambulance service. It had been said that the people in an English town had the health and hospital provision which they deserve. As he read the times, the patience of Londoners was almost, if not quite, exhausted so far as the neglect of the County Council in its treatment of the ambulance and health questions was concerned. Either this body must promptly do its duty and act up to the pledges of many of its members before they were elected to the Council, or the people may suddenly be aroused to protect themselves by destroying the party system. Then they would speedily secure the accommodation which they ought to have had long ago, had it not been for the supineness of a majority of the men who have got themselves elected to the London County Council.

